# Cervical cancer screening by cytology and the burden of epithelial abnormalities in low resource settings: a tertiary-center 42-year study

**DOI:** 10.1186/s12905-024-03254-1

**Published:** 2024-07-17

**Authors:** Sahar Ezzelarab, Amro El-Husseiny, Magda Nasreldin, Radwa Ali, Ashraf Nabhan, Early Cervical Cancer Detection Consortium, Maya Abdel-Razek, Magda Abdel-Salam, Reem Abdel-Salam, Rania Ahmed, Amal Alloub, Hamdy Alqenawy, Amal Alshabrawy, Nahla Awad, Sohair Ayaad, Hala El-Sallaly, Mortada El-Sayed, Ragia Fahmy, Asmaa Kasem, Ghada Moubarak, Hasan Morsi, Ragaa Rifaat, Noha Sakna, Zeinab Shehabeldin, Ahmed Zenhom

**Affiliations:** Department of Obstetrics and Gynecology, Faculty of Medicine, Ain Shams University, Abbassia, Cairo, 11566 Egypt

**Keywords:** Pap smear, Early detection of cancer, Cancer screening, Uterine cervical neoplasms, Cervical cancer, Squamous intraepithelial lesions of the cervix

## Abstract

**Background:**

Cytological screening remains a high-impact practice, particularly in low-resource settings, for preventing cervical cancer. The examination of screening practices over time and the prevalence of epithelial abnormalities have not been investigated in longitudinal studies in one of the largest countries in the Middle East and Africa.

**Methods:**

Routine healthcare data, between March 1981 and December 2022, were extracted from the database of the Early Cancer Detection Unit in a tertiary referral university hospital in the Greater Cairo Region, Egypt. Cervical smears were obtained using a standardized technique and sent to the cytopathology laboratory for conventional cytology examination by expert pathologists. The anonymous data were analyzed to determine the temporal trend of the number of women screened each year and the prevalence of epithelial abnormalities.

**Results:**

Data included the results of satisfactory smears from 95120 women. The mean age (SD) of the women at the time of screening was 38.5 (10.5). None of the included women received an HPV vaccine. Abnormal epithelial cells were reported in 5174 women (5.44%). Of these epithelial abnormalities, the majority were low-grade squamous intraepithelial lesions in 4144 women (4.36%). Other abnormalities included atypical squamous cells in 378 women (0.40%), high-grade squamous intraepithelial lesions in 226 women (0.24%), atypical glandular cells not otherwise specified in 184 women (0.19%), adenocarcinoma in 165 women (0.17%), squamous cell carcinoma in 70 women (0.07%), and atypical glandular cells favoring neoplasms in 7 women (0.01%). Women who were at an early age at first intercourse, those who opted for routine cervical cytology screening, and those who were older at screening were more likely to have epithelial abnormalities. The yearly number of screened women was positively associated with the detection of low-grade squamous intraepithelial lesions (correlation coefficient [95% CI] = 0.84 [0.72, 0.91]) and negatively associated with the detection of squamous cell carcinoma (correlation coefficient [95% CI] = -0.55 [-0.73, -0.29]).

**Conclusions:**

The small number of annually screened Egyptian women and the temporal trend in epithelial abnormalities critically demonstrate the need for establishing and scaling up a structured population-based program to achieve the goal of eliminating cervical cancer.

## Background

Cervical cancer, a matter of public health concern, is the fourth most prevalent form of cancer in women worldwide and ranks as the 14th in Egypt [1].

The natural history of cervical cancer has a multistep carcinogenesis model, wherein the critical stages are Human Papilloma Virus (HPV) infection, progression to a precancerous state, and subsequent development of invasive cancer [2, 3].

The incidence of cervical cancer, a largely preventable cancer, has decreased over the past few decades in most countries, although the extent of this reduction varies. HPV vaccination and screening for precursor lesions, followed by appropriate follow-up and treatment contribute to this reduction [4, 5]. This laid the foundation for the World Health Organization (WHO) global initiative to eliminate cervical cancer. One cornerstone of this initiative aims at achieving a target of 70% screening coverage of women aged 30–49 years with a high-performance test [6].

Screening forms the initial mandatory step of the two approaches towards the prevention of cervical cancer, whether the screen-and-treat approach or the screen, triage and treat approach [4].

Screening is available in many countries through either population-based (organized) or non-population-based (unorganized) programs, as well as through opportunistic screening. Participation rates and coverage vary significantly across countries and settings. The key factors influencing participation are socioeconomic status, health insurance coverage, public awareness, and level of education. Additionally, some women may face barriers to accessing these services due to a lack of power, authority, or control, which can be major obstacles to their participation [7–9].

According to the 2015 Egypt Health Issues Survey, the knowledge and practice of Pap smears for cervical cancer screening among women are quite limited. Only 7 percent of women between the ages of 15 and 59 had ever heard of a pap smear, and a very small percentage (0.3 percent) had undergone the procedure. The proportion of women who were aware of pap smears exceeded 10 percent only among women residing in urban governorates (14 percent), women working for cash (13 percent), and women in the highest wealth quintile (11 percent). In all subgroups, one percent or less of women reported ever having had a Pap smear [10].

Given the limited knowledge and low practice of cervical screening among Egyptian women, coupled with the absence of a national population-based screening program, there is a lack of comprehensive understanding regarding the trends of epithelial abnormalities in cervical cytology within the largest population in Africa and the Middle East. Previous research has not thoroughly investigated the outcomes of cervical cancer screening initiatives among Egyptian women or how they evolve over time. Therefore, our objective was to examine the temporal trends in cervical cytology practice and the observed epithelial abnormalities.

## Methods

This retrospective chart review used routinely collected health data from the Early Cancer Detection Unit (ECDU) following Institutional Review Board approval from the Department of Obstetrics and Gynecology, Ain Shams University, Cairo, Egypt.

### Study population and data source

Study population included adult women who attended Ain Shams University Hospital of Obstetrics and Gynecology, whether in the ECDU unit, Gynecologic outpatient clinic, or inpatient and were willing to have a cervical cytology for the first time. The database did not include women who were pregnant, menstruating, known to have invasive or preinvasive cervical cancer, or a prior cervical surgery.

The ECDU began opportunistic screening and treatment for cervical cancer in March 1981. The ECDU provides the service at a subsidized cost that is much cheaper than women would find at other healthcare facilities in Egypt. Participants included all adult women who did not have any prior screening in the past 10 years and who underwent conventional cervical screening in the ECDU from March 1981 to December 2022.

No formal referral process exists between healthcare facilities in Egypt. However, the resources available at the ECDU for the detection and treatment of cervical cancer make screening at the ECDU an essential step in the continuum of cancer prevention and control within the country. As a result, many women are referred to the ECDU from public or private healthcare facilities across Egypt to obtain a pap smear to obtain follow-up evaluations after a suspicious screening using other methods, such as visual inspection aided by acetic acid or Lugol’s iodine, or to begin the process of cancer treatment. The ECDU does not employ a systematic method of recruiting women to undergo cervical cancer screening; therefore, the women were either self-referred or referred by a healthcare provider for screening. Data identifying the source of referral for screening as the individual or a healthcare provider were not captured in this dataset. According to ECDU institutional practice, women who have low-grade infections at the time of a Pap smear are screened again within 6 months of the prior visit, indicating that women often have multiple screening visits.

The ECDU started to use an electronic database in 1986, in addition to paper records, to capture relevant demographics (i.e., age, address, marital status, number of pregnancies), risk factors for cervical cancer (i.e., previous sexually transmitted infections, parity, oral contraceptive use), and clinical outcomes (outcomes of Pap smear) of women who were screened at the clinic. In the first Pap smear, a unique identifying number was used to update each woman’s record. The database has been maintained since its inception, making it a reliable source of longitudinal data for examining trends in cervical cancer screening in a tertiary university healthcare facility in Egypt. All paper records, prior to the establishment of the electronic records system, were transcribed into a database.

Cervical cytology (Pap smear) was obtained using an Ayer spatula and spread over a marked glass slide, which was placed in 95% ethyl alcohol and sent to the cytopathology laboratory for conventional cytology examination. The data were recorded using a structured form. The results of the examined smears were reported according to the Bethesda System for Reporting Cervical Cytology. The terminology “CIN1”, “2”, and “3” was used in the ECDU until 2004. Following the adoption of the Bethesda system, the ECDU transformed all the data to match the new system terminology.

### Data analyses

The ECDU provided a deidentified dataset spanning a 42-year period. We used this dataset to describe the clinical and demographic characteristics of screened women, to examine longitudinal trends in screening practice and to report trends in epithelial abnormalities.

Epithelial abnormalities captured at screening were categorized into normal results, low-grade abnormalities (Atypical Squamous Cells of Undetermined Significance or AS-CUS, and Low-grade Squamous Intraepithelial Lesion or LSIL classifications), and high-grade abnormalities (Atypical Squamous Cells, High-grade Lesion or ASC-H, Atypical Glandular Cells or AGUS, High-grade Squamous Intraepithelial Lesion or HSIL, and Invasive Cancer). The Bethesda system of classification of cervical smears has evolved over the time period in this study. We have re-categorized results to fit the updated classification where possible. In an effort to maintain consistency of our reported results, we have transitioned our old cytology results to the comparable categories in the Bethesda system. This change allowed us to present all our statistics in a consistent and standardized format, making it easier to track trends and identify insights. The reports of moderate and severe dysplasia were grouped in the group of HSIL. Mild dysplasia and HPV infection were grouped as LSIL.

We summarized participants’ characteristics, including age at first screening, reporting means and standard deviations. We summarized Pap smear results by the Bethesda classification system as categorical variables, reporting proportions for each variable. The primary outcome of interest was the number of pap smears completed by year, including the cytological results of those screenings.

The data were analyzed using the Statistical Package for the Social Science (SPSS), Version 20 (IBM Corp., Armonk, USA).

## Results

Over a 42-year period from March 1981 to December 2022, 100155 Pap smears were collected from women attending the ECDU at the Department of Obstetrics and Gynecology, Ain Shams University, Cairo, Egypt. Following data cleaning, 95120 smears were obtained for data analysis.

The mean age of the women screened was 38.48 years (10.45). The mean age of first intercourse was 20.05 years (1.39). Most women were married and were examined routinely Table 1.

**Table 1:** Demographic characteristics of women screened, 1981– 2022

Demographic and clinical characteristics	Mean (SD) or number (%)	Valid number
Age at screening (years)	38.48 (10.45)	95120
Age at first intercourse (years)	20.05 (1.39)	89490
Marital status		95120
Married	93709 (98.52)	
Widow	819 (0.86)	
Divorced	506 (0.53)	
Separated	86 (0.09)	
Indication for screening		95120
Not reported	53354 (56.09)	
Symptomatic	30352 (31.91)	
Routine asymptomatic	11414 (12.00)	

Among women who opted for screening due to clinical symptoms, vaginal discharge was the most common symptom to warrant a smear.

Most smears (57072/95120 [60.00%]) were obtained following referral from a healthcare provider to the ECDU.

**Fig. 1:**
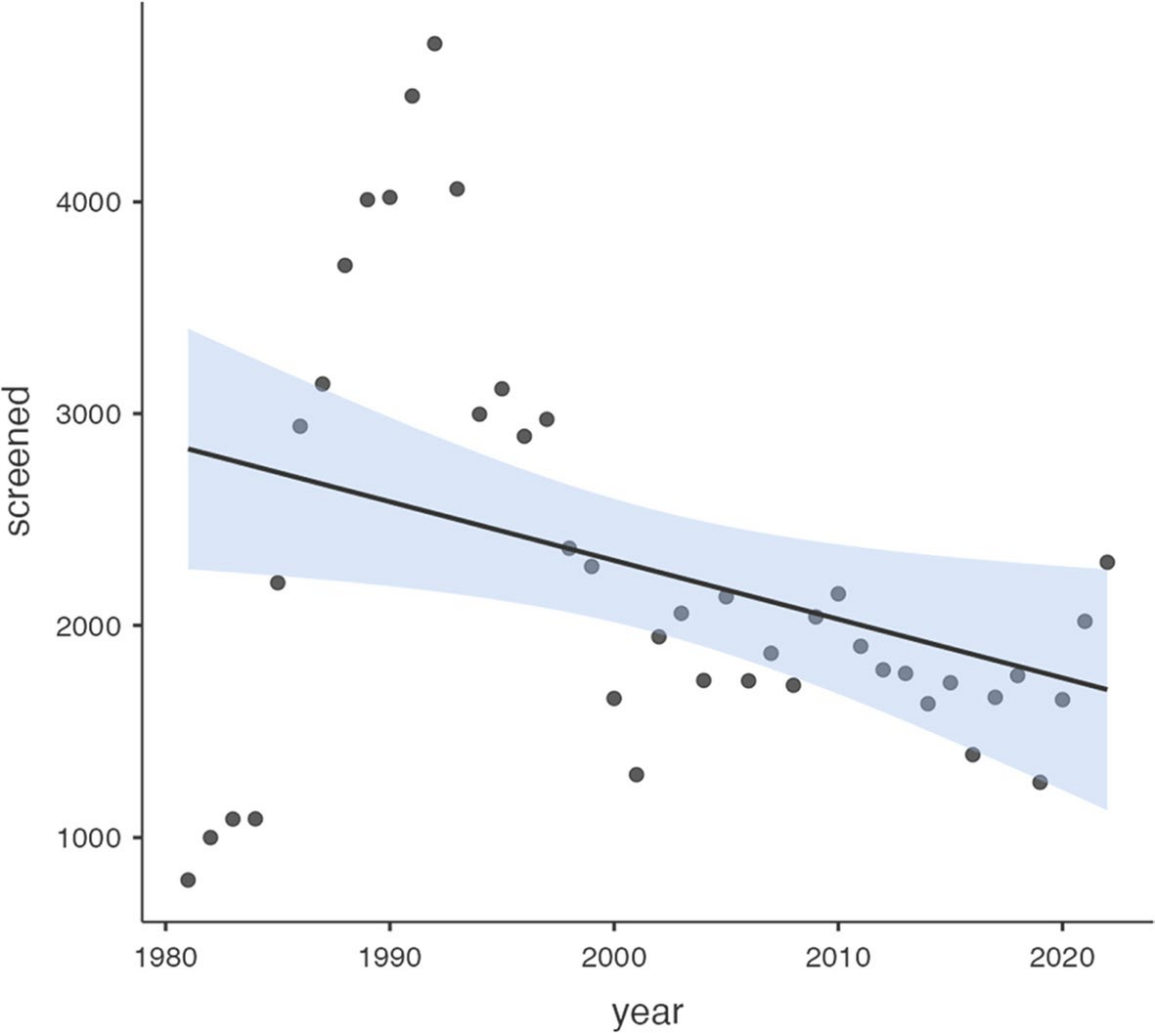
Temporal trend of the number of screened women by year at ECDU, Ain Shams University, 1981– 2022

The temporal trend of screening is depicted in Fig. 1. The median [IQR] number of women who underwent cervical cytology screening was 1982 [1674, 2928].

Epithelial abnormalities were absent in 94.56% of women (89946/95120; with or without inflammatory cells 71971 and 17975, respectively). Pap smears revealed epithelial abnormalities in 5.44% of the participants (5174/95120). Epithelial cell abnormalities included LSIL (low-grade squamous intraepithelial lesion) in 4144 women (4.36%) (condyloma in 3113 (3.27%) and CIN I in 1031 (1.08%) women), atypical squamous cell (ASC) in 378 (0.40) (ASCUS in 351 (0.37%) and ASC-H in 27 (0.03%) women), HSIL (high-grade squamous intraepithelial lesion) in 226 (0.24%) (HSIL-CIN II in 202 and HSIL-CIN III in 24 women), and squamous cell carcinoma in 70 (0.07%) women. There were 184 women (0.19%) with atypical glandular lesions not otherwise specified and 165 women (0.17%) with adenocarcinoma (malignant endocervical cells in 155 and malignant endometrial cells in 10 women), Table 2.

**Table 2:** Results of cervical cytology among women screened, 1981– 2022

Finding	Frequency	Relative frequency
NILM	89946	94.56%
LSIL	4144	4.36%
ASC	378	0.40%
HSIL	226	0.24%
AG cells NOS	184	0.19%
Adenocarcinoma	165	0.17%
Malignant SCC	70	0.07%
AG cells favor neoplastic	7	0.01%
Total	95120	100.00%

During the period from 1981 to 2004, the percentage of condylomas decreased from 4 to 1%, and that of CIN I decreased from 9% to 0.52%.

The detection of LSIL decreased from 59/800 (7.38%) in 1981 to 103/2298 (4.48%) in 2022, while that of HSIL decreased from 10/800 (1.25%) to 3/2298 (0.13%).

**Fig. 2:**
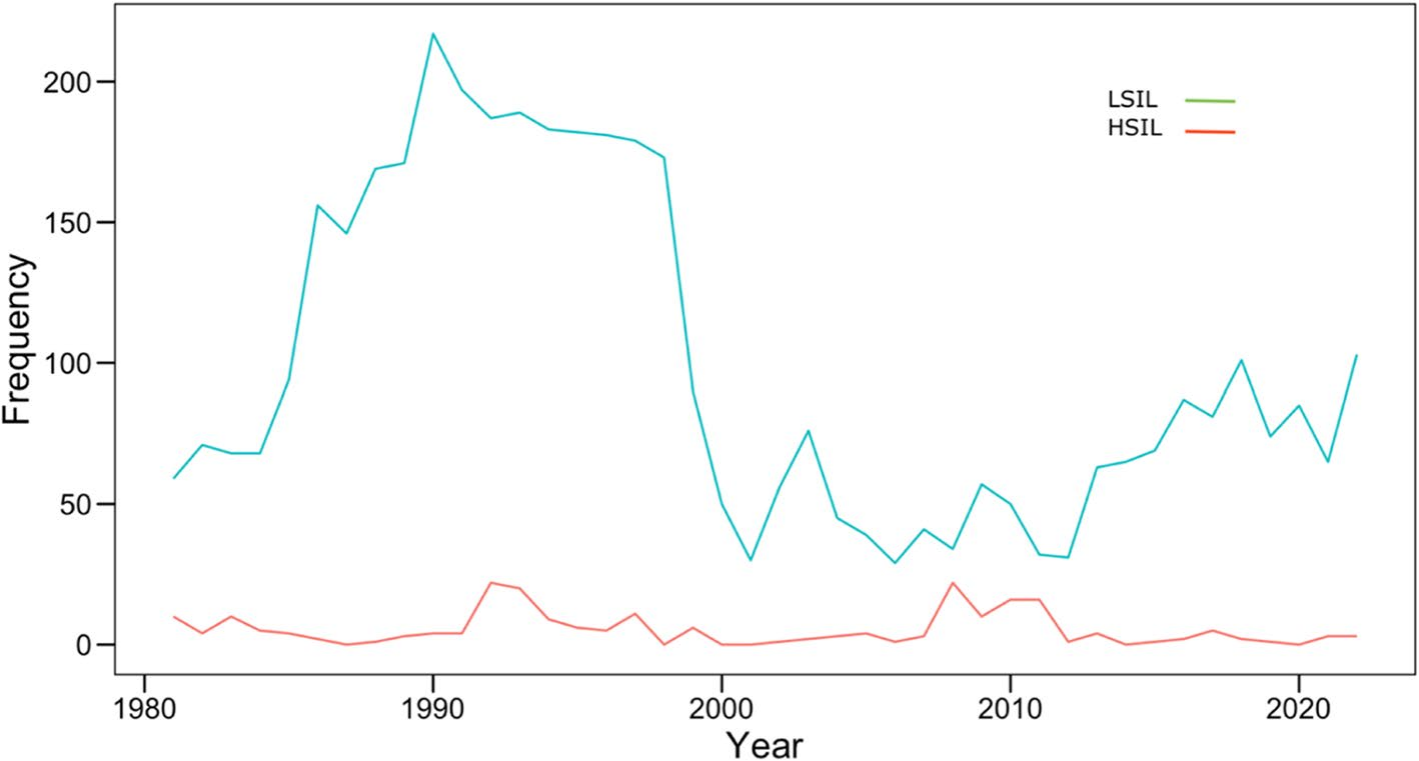
Temporal trend of squamous intraepithelial lesion by year among women screened at ECDU, Ain Shams University, 1981– 2022

**Fig. 3:**
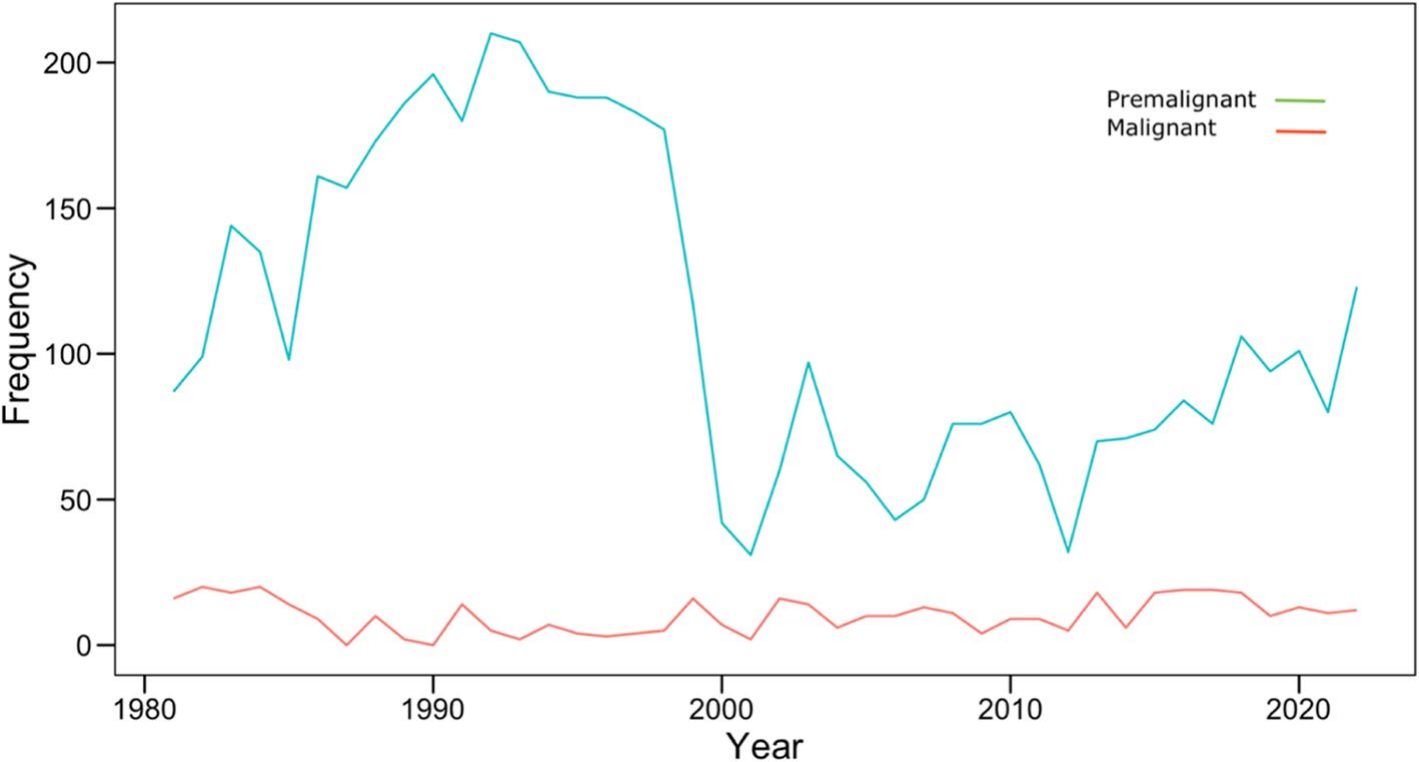
Temporal trend of premalignant and malignant findings by year among women screened at ECDU, Ain Shams University, 1981– 2022

Preinvasive lesions (ASC, LSIL, and HSIL) declined from 87/800 (10.88%) in 1981 to (123/2298 (5.35%) in 2022. The temporal trends are depicted in Fig. 2 and Fig. 3.

Patients with abnormal cells had a significantly older mean age than those with normal cells (40.5 versus 38.4 years, *P* < 0.001). Patients with ASC were significantly older than those with LSIL and younger than those with HSIL, and there were more patients with glandular cells (favor neoplastic) (*P* < 0.001). The mean age of patients with LSILs was significantly younger than that of patients with other abnormal cells (*P* < 0.001). Patients with LSILs and AGUS were significantly younger than patients with malignant squamous cell carcinoma, HSILs or adenocarcinoma (*P* < 0.05).

Epithelial abnormalities increased significantly with each decade of age, Table 3.

**Table 3:** Results of cervical cytology by age groups among women screened from 1981 to 2022

Age groups in years		≤ 20	21–30	31–40	41–50	51–60	> 60
Pap Smears	**89946**	1656	22994	33614	25586	8700	2570
Abnormal cells	**5174**	57	976	1717	1611	575	238
Proportion	**3.4%**	3.4%	4.2%	5.1%	6.3%	6.6%	9.3%
*P* value OR (95%CI)			0.13 1.24 (0.95–1.63)	**0.002** 1.51 (1.15–1.98)	** < 0.001** 1.88 (1.44–2.47)	** < 0.001** 1.99 (1.50–2.62)	** < 0.001** 2.86 (2.13–3.85)
			*	** < 0.001** 1.21 (1.12–1.32)	** < 0.001** 1.52 (1.40–1.64)	** < 0.001** 1.60 (1.44–1.78)	** < 0.001** 2.30 (1.99–2.67)
				*	< 0.001 1.25 (1.16–1.34)	** < 0.001** 1.31 (1.19–1.45)	** < 0.001** 1.90 (1.65–2.19)
					*	0.30 1.05 (0.95–1.16)	** < 0.001** 1.52 (1.32–1.75)
						*	** < 0.001** 1.44 (1.23–1.69)

Chi-square test

*OR* Odds ratio, *95%CI* 95% confidence interval

^*^Group against which comparisons were performed

Epithelial abnormalities were significantly more common in women who underwent routine check-ups than in symptomatic women (OR [95% CI] 1.2 [1.11, 1.30], *P* < 0.001). Women with epithelial abnormalities were significantly younger at first intercourse (*P* < 0.001).

**Fig. 4:**
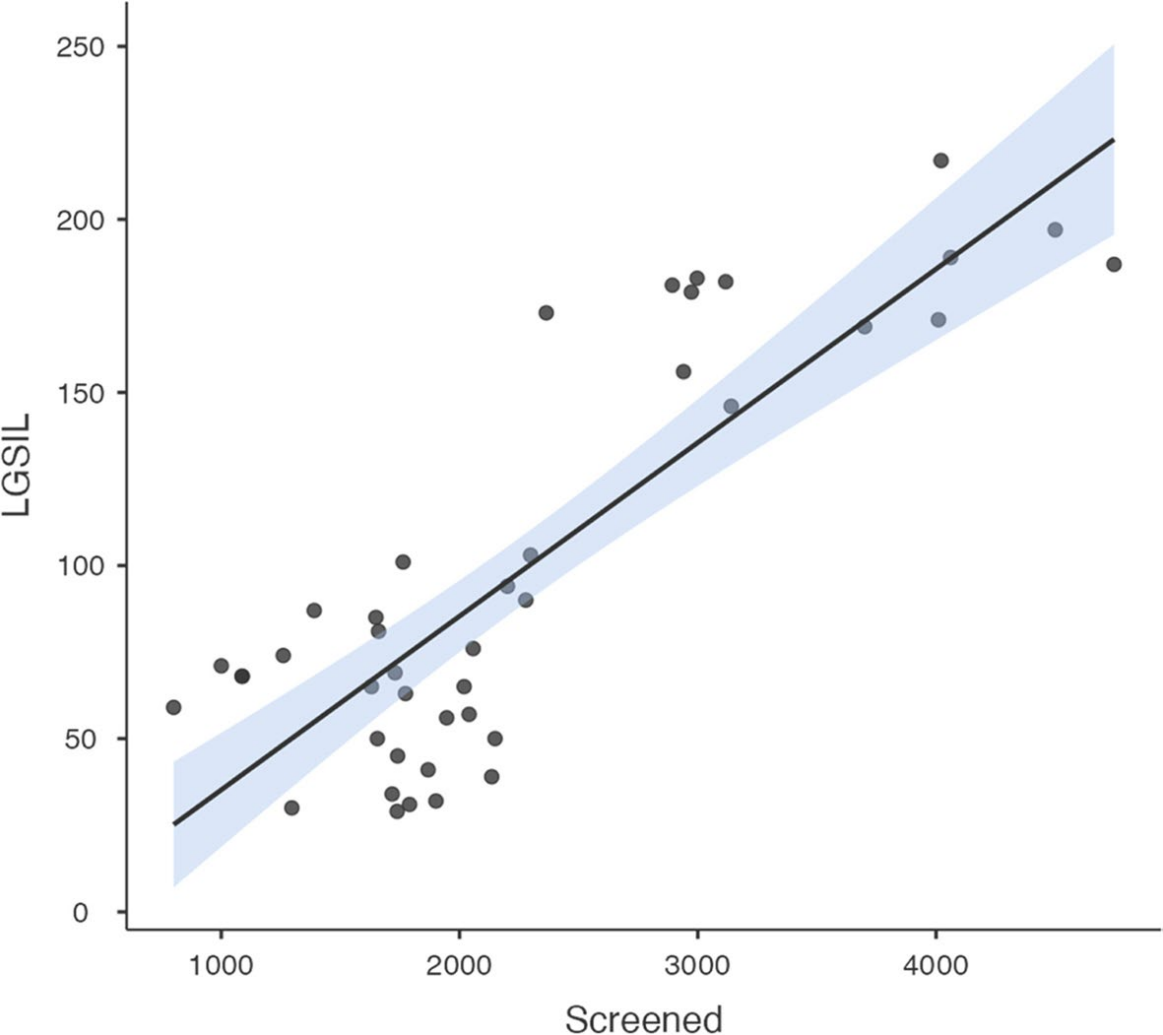
Yearly screened women and LSIL

**Fig. 5:**
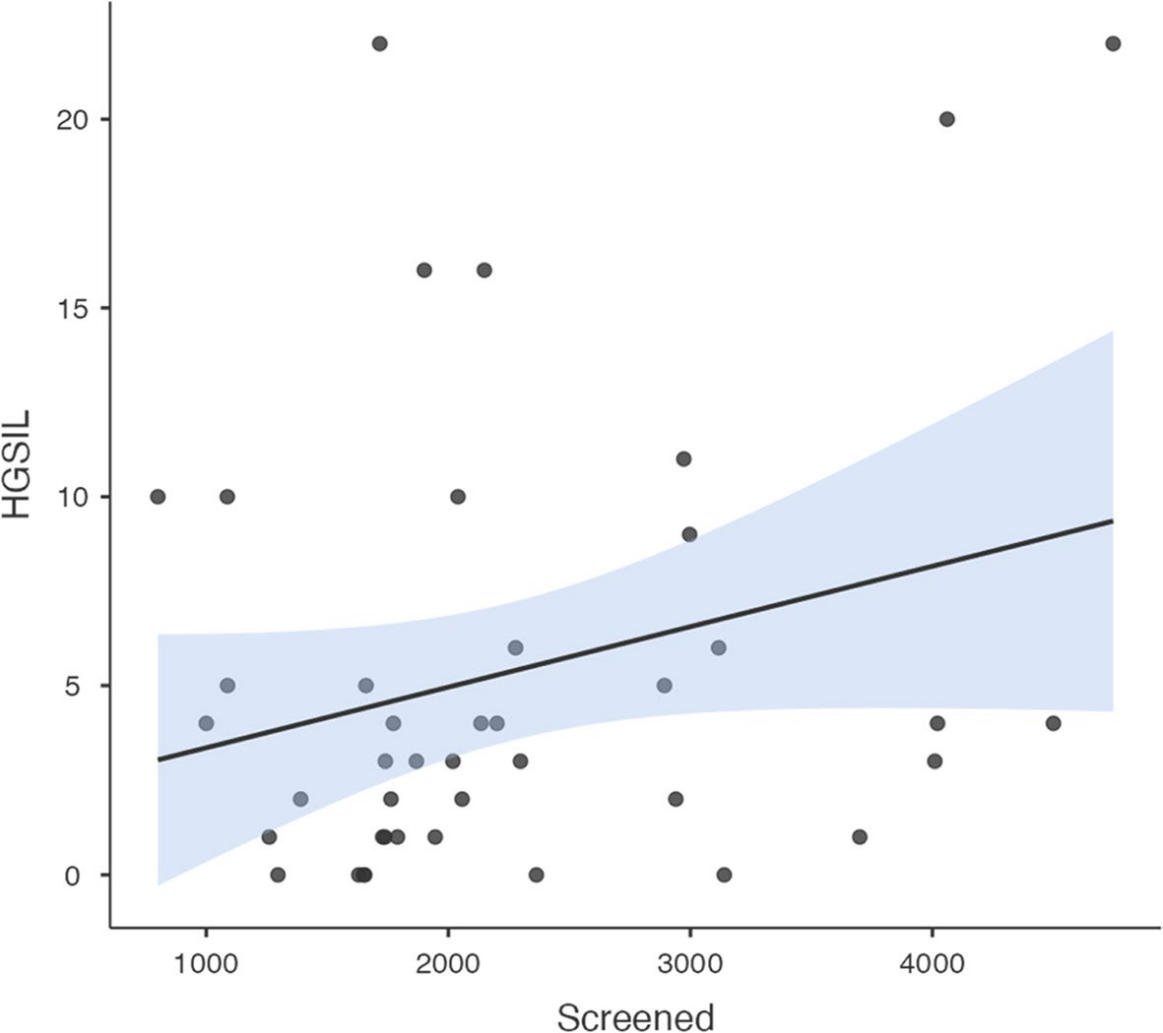
Yearly screened women and HSIL

**Fig. 6:**
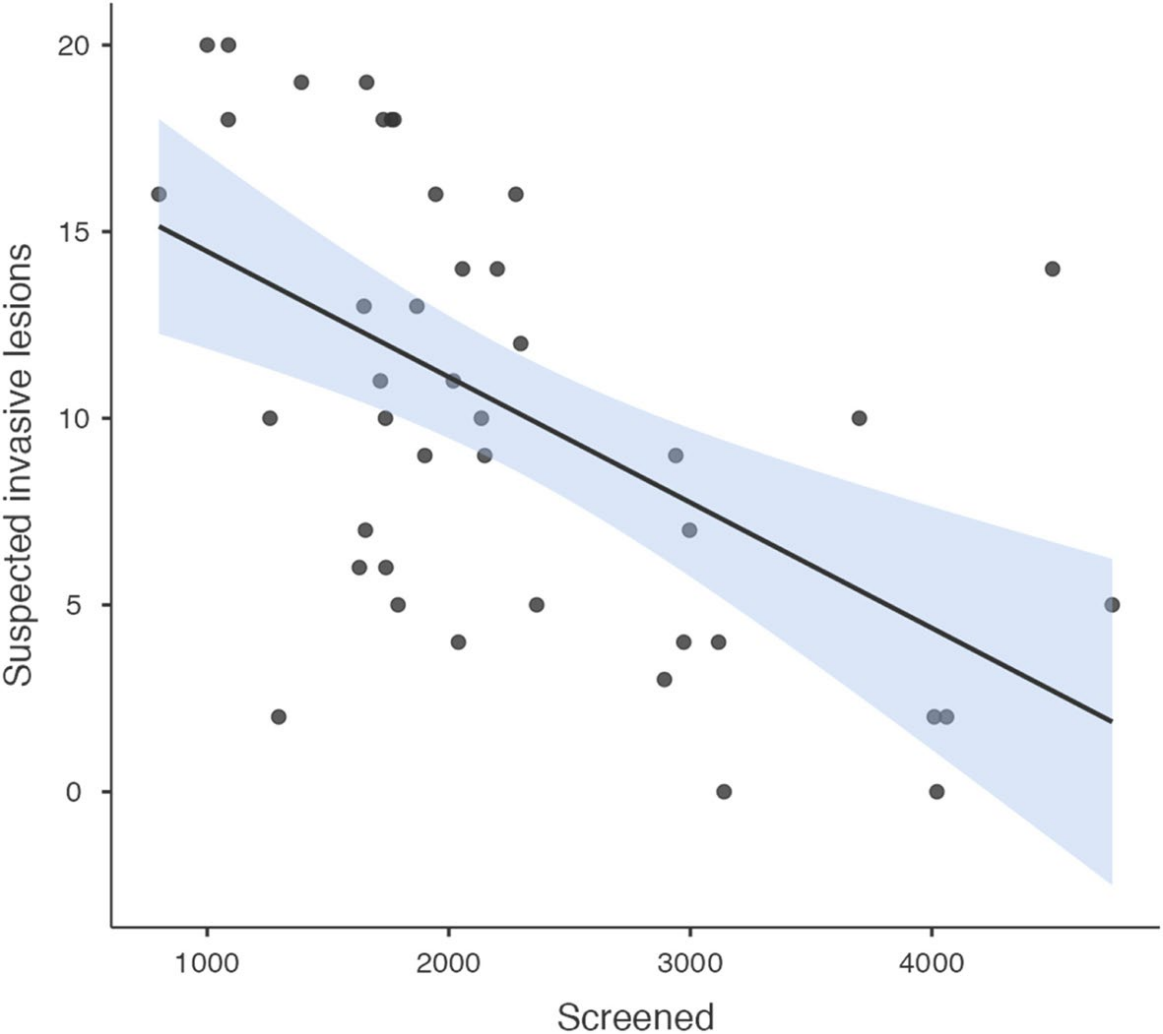
Yearly screened women and suspected invasive lesions

The yearly number of screened women was positively associated with the observed LSIL (correlation coefficient [95% CI] = 0.84 [0.72, 0.91]) (Fig. 4), not significantly associated with the HSIL (correlation coefficient [95% CI] = 0.26 [-0.05, 0.52]) (Fig. 5), and negatively associated with the observed suspected invasion (correlation coefficient [95% CI] = -0.55 [-0.73, -0.29]) (Fig. 6).

## Discussion

### Key results

By analyzing routinely collected healthcare data from 95,120 satisfactory cervical smear records in the ECDU, this study reported trends in screening uptake and in the observed epithelial abnormalities. A notable increase in the number of annually screened women occurred from 1981 to 1992, after which the number of screened women declined. Women who engaged in sexual intercourse at a young age and who made the conscious decision to undergo routine cytological screening exhibited a greater probability of presenting with epithelial abnormalities. Furthermore, these abnormalities were more prevalent among women who underwent the screening process at a relatively advanced age than among their counterparts. The most common epithelial abnormality was LSILs. The yearly number of cervical screenings by cytology was positively associated with LSIL and negatively associated with invasive lesions.

The discrepancy between the recommended cervical cancer screening guidelines and actual clinical practice was identified in a recent systematic review. Only six of 11 countries across North America, Europe, and the Asia–Pacific region have implemented comprehensive population-based screening programs [11]. This observation highlights a potential gap between policy recommendations and real-world implementation of cervical cancer prevention strategies.

Current consensus does not recommend the initiation of cervical cancer screening before the age of 21 years in immunocompetent females due to the very low rate of cervical cancer among women aged 20 to 24 years (0.8 per 100,000) [12]. In Egypt, there is a social tendency for early marriage, and the results of this study revealed that epithelial abnormalities are associated with earlier sexual activity. This might have implications for practice.

This study revealed a 5.44% prevalence of epithelial abnormalities in cervical smears. This finding aligns with reports from other Arab countries, such as Saudi Arabia (4.27%) [13], Jordan (3.8%) [14], the United Arab Emirates (3.3%) [15], and Kuwait (4.4%) [16]. This similarity might be related to shared cultural and religious factors. This prevalence in our study is much lower than that reported in sub-Saharan Africa, as reported in studies from southwestern Nigeria (34.6%) [17] and Northwest Ethiopia (14.1%) [18]. Coexisting HIV infection in these regions is a potential explanation for the higher rates.

In the current study, epithelial abnormalities were significantly more common in women who underwent routine check-ups than in symptomatic women. Other reports have not found such a significant difference [13]. This finding can provide support to the cervical cancer control efforts by providing evidence on the benefit of routine screening rather than only when symptoms arise.

In an age-stratified analysis of the distribution of cytological abnormalities, we found that women older than 60 had the highest prevalence of epithelial abnormalities, possibly due to the limited organized screening programs in Egypt in younger women with accumulation of epithelial abnormalities over time.

Our results indicate that women with ASC were significantly older than those with LSIL and younger than those with HSIL and glandular cells. Patients with LSILs and AGUS were significantly younger than patients with malignant squamous cell carcinoma, HSILs or adenocarcinoma. This was consistent with the findings of previously published results showing that the incidence of ASC, LSIL, and HSIL peaked in the 30–39-year age group, while the incidence of AGUS peaked among individuals aged 40–49 years. The incidence of malignant lesions further increased after the age of 50 years. The mean ages at diagnosis for patients with LSILs and HSILs were 34.7 and 37.7 years, respectively, while patients with malignant lesions presented with a mean age at diagnosis of 51.8 years [19]. This might have implications for screening practice [20].

Over a 42-year period, the number of screened women has decreased. This would explain the decrease in the number of SIL abnormalities from 6.9% to 4.3% for LSILs and from 1.2% to 0.13% for HSILs. This is because screening services in ECDUs currently operate on an opportunistic basis rather than through a structured, population-based approach.

The effectiveness of structured, population-based preventive strategies in reducing the incidence rates of preinvasive and invasive lesions of the cervix cannot be overstated [21–24]. However, in the vast majority of low- and middle-income countries (LMICs), including Egypt, researchers have identified a diverse array of obstacles to the process of screening [25]. LMICs need prompt and immediate execution of unambiguous regulations, which should be fortified by the ability of the healthcare system to put these regulations into action. We need widespread advocacy within the community and the dissemination of information, alongside the strengthening of policies that promote the well-being of women and ensure gender equality.

### Strengths and limitations

Overall, a four-decade study of cervical screening practice in a low resource setting, offers a wealth of information that can significantly contribute to the understanding and improvement of cervical cancer prevention and control. The extended timeframe allows for the collection of comprehensive data providing insights into trends and changes in cervical screening practices. Understanding how cervical screening practices have evolved over four decades can highlight the impact of policy changes and public health initiatives on screening rates. Highly competent pathologists assessed and confirmed cytologic findings, reducing the chances of incorrect classification of screening results. The results obtained in this study, when interpreted in the context of clinical practice, reveals the actual practice in the largest facility in a country of lower middle income and shows the urgent need to adopt a structured screening program and to incporporate state of the art tools for the diagnosis and management [26, 27]. The extensive dataset collected can serve as a valuable resource for future research, allowing for more detailed analyses and the exploration of new research questions.

Limitations of this work includes missing data especially in certain demographic characteristics of women. This might reflect sensitive data in a conservative community or the lack of rigor or inconsistencies when collecting routine data. All demographic data were self-reported by women, which is prone to recall bias. The process of screening for cervical cancer is characterized by opportunistic practices rather than organized efforts. This implies that the subset of women who undergo screening differs from those who do not, as the former group has successfully surmounted various obstacles, such as financial constraints, social factors, cultural influences, and geographic limitations, to avail themselves of screening services. There is also a lack of information relating to the source of referral (e.g., self-referred or provider referred) for screening. The change in terminology used over time may have some implications for the assigned category of abnormality observed. The study lacks the findings on repeated smears for abnormal cytology.

HPV testing became a state of the art in the screening and management [26, 28]. However, the current study did not examine how changes in the healthcare system, such as the introduction of new technologies (e.g., HPV testing), have affected cervical screening practices because of the lack of affordable and accessible and HPV testing nationwide.

This four-decade longitudinal study could have highlighted the role of preventive measures, such as the HPV vaccine, in reducing the incidence of cervical cancer. Unfortunately, the vaccination coverage is unknown and there is no national vaccination program.

## Conclusion

The findings can inform public health policy and help in the allocation of resources to improve screening programs and implement effective preventive measures. The findings clearly show the need for a population-based national program if we are keen to meet the World Health Organization’s 2030 targets for cervical cancer elimination.

## Data Availability

All data relevant to this study are publicly available. The data, analysis script and materials related to this study are publicly available on an open access registry. To facilitate reproducibility, this manuscript was written by interleaving regular prose and analysis code using R Markdown. The integrity of raw data is the responsibility of the first author Prof. Sahar Ezzelarab.

## Abbreviations

AG-US: Atypical glandular cells of undermined significance
ASC: Atypical squamous cell
ASC-H: Atypical squamous cell-induced HSILs cannot be excluded.
ASC-US: Atypical squamous cell of undetermined significance
CIN: Cervical intraepithelial neoplasia
ECDU: Early Cancer Detection Unit
HSIL: High-grade intraepithelial lesion
HPV: Human papillomavirus
LSIL: Low-grade squamous intraepithelial lesion
NILM: Negative for intraepithelial lesion or malignancy
NOS: Not otherwise specified
SCC: Squamous cell carcinoma
